# Dioscin Induces Apoptosis in Human Cervical Carcinoma HeLa and SiHa Cells through ROS-Mediated DNA Damage and the Mitochondrial Signaling Pathway

**DOI:** 10.3390/molecules21060730

**Published:** 2016-06-04

**Authors:** Xinwei Zhao, Xufeng Tao, Lina Xu, Lianhong Yin, Yan Qi, Youwei Xu, Xu Han, Jinyong Peng

**Affiliations:** College of Pharmacy, Dalian Medical University, Western 9 Lvshunnan Road, Dalian 116044, China; zhaoxinwei2015dy@163.com (X.Z.); taoxufeng@dmu.edu.cn (X.T.); Linaxu_632@126.com (L.X.); Lianhongyin_1980@163.com (L.Y.); Yanqi_1976@163.com (Y.Q.); Youweixu_1964@163.com (Y.X.); Xuhan2002zs@163.com (X.H.)

**Keywords:** apoptosis, dioscin, DNA damage, ROS, cervical carcinoma

## Abstract

Dioscin, a natural product, has activity against glioblastoma multiforme, lung cancer and colon cancer. In this study, the effects of dioscin against human cervical carcinoma HeLa and SiHa cells were further confirmed, and the possible mechanism(s) were investigated. A transmission electron microscopy (TEM) assay and DAPI staining were used to detect the cellular morphology. Flow cytometry was used to assay cell apoptosis, ROS and Ca^2+^ levels. Single cell gel electrophoresis and immunofluorescence assays were used to test DNA damage and cytochrome C release. The results showed that dioscin significantly inhibited cell proliferation and caused DNA damage in HeLa and SiHa cells. The mechanistic investigation showed that dioscin caused the release of cytochrome C from mitochondria into the cytosol. In addition, dioscin significantly up-regulated the protein levels of Bak, Bax, Bid, p53, caspase-3, caspase-9, and down-regulated the protein levels of Bcl-2 and Bcl-xl. Our work thus demonstrated that dioscin notably induces apoptosis in HeLa and SiHa cells through adjusting ROS-mediated DNA damage and the mitochondrial signaling pathway.

## 1. Introduction

Cervical carcinoma, a malignant gynecological disorder, is a common cause of death in females. The cervical cancer mortality rate ranks fourth among all kinds of cancers in China, and the second in gynecological cancers. Human papillomavirus (HPV) infection is the most important cause of cervical cancer, with more than 99% of cervical carcinomas testing positive for human HPV DNA [1].

At present, the methods to treat cervical cancer mainly include surgery, radiotherapy and integrated Chinese and Western medicines. Cervical cancer is not sensitive to most cancer drugs, which results in low chemotherapy efficiency (<15%). Patients can be treated by combining chemotherapy and radiotherapy. Platinum-based chemotherapeutical drugs have been proven to be effective to treat cervical cancer in the clinic, however, these chemical drugs cause a large number of serious side effects, including nephrotoxicity, ototoxicity, neurotoxicity, gastrointestinal reactions and bone marrow toxicity. Thus, the development of novel potential drug candidates with high efficacy and low side effects for treating cervical cancer is very urgent.

Traditional Chinese medicines (TCMs) with low toxicity and high efficacy have been used to treat cancers for thousands of years [2] and are getting more and more attention in recent years. Some natural products, including vincristine [3,4] and camptothecin [5], have been used for the clinical treatment of cancers. Hence, it is reasonable to seek new and effective natural products to treat cervical cancer among TCMs.

**Figure 1:**
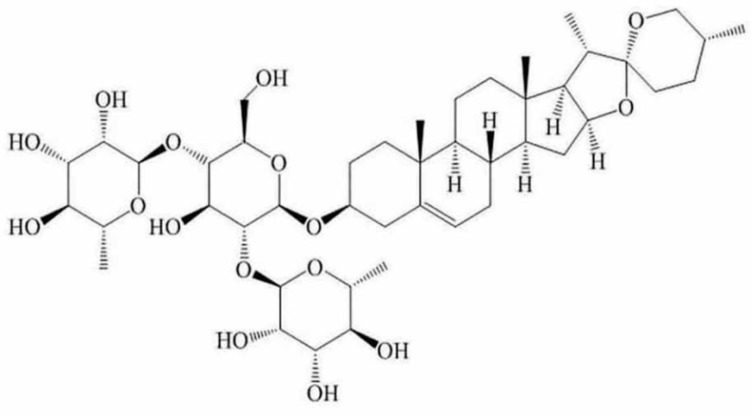
The chemical structure of dioscin.

A growing volume of research suggests that steroidal saponins have wide pharmacological effects and biological activities, including anti-tumor, anti-fungal, cardiovascular diseases preventing and treating, hypoglycemic and immune regulation effects. Among them, diosgenin, polyphyllin and OSW-1 all possess potent anti-cancer activities [6,7,8,9]. Dioscin (Figure 1), a typical steroidal saponin, is mainly derived from medicinal plants including *Dioscorea nipponica* Makino and *Dioscorea zingiberensis* Wright. Pharmacological studies have shown that dioscin has anti-fungal [10], anti-virus [11], hepatoprotective [12,13] and anti-cancer activities [14]. Up to now, we already know that dioscin can induce the apoptosis of HeLa cells [15]. However, to our best knowledge, there have no published papers to report the anti-cancer effects of dioscin against SiHa cells, and the mechanisms of the action on cervical cancer are still unknown. Therefore, the aim of the present study was further to confirm the effects of dioscin against HeLa and SiHa cells *in vitro*, and then to investigate the possible mechanisms.

## 2. Results

### 2.1. Dioscin-Inhibited Cell Viability

**Figure 2:**
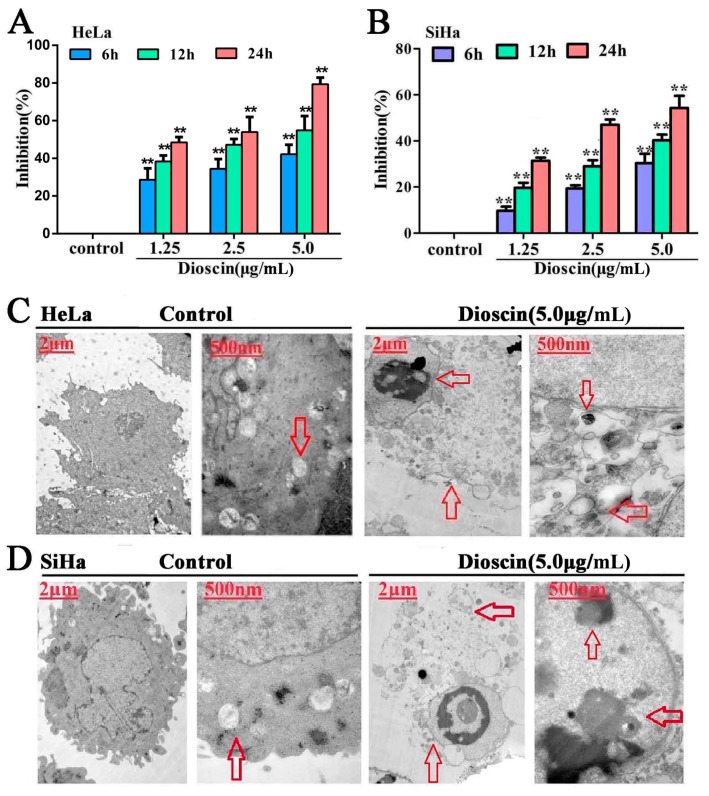
Dioscin inhibited cell viability and induced ultrastructure changes. (**A**) Inhibition effects of dioscin on HeLa cells; (**B**) Inhibition effects of dioscin on SiHa cells; (**C**) Effects of dioscin on the ultrastructure of HeLa cells by TEM assay; (**D**) Effects of dioscin on the ultrastructure of SiHa cells by TEM assay. Data are presented as mean ± SD (*n* = 5). ** *p* < 0.01 compared with control group. The images of the cells gestalt structures (6000×, final magnification).

In the present paper, the MTT assay results showed that the proliferation of HeLa and SiHa cells was significantly inhibited by dioscin in a time- and dose-dependent manner. Especially for HeLa cells (Figure 2A), compared with control groups, the inhibition rates when treated by 5.0 μg/mL of dioscin for 12 h and 24 h were 47.79% and 80%.Thus, HeLa cells treated with dioscin at concentrations of 1.25, 2.5 and 5.0 µg/mL for 12 h were selected for subsequent experiments. Compared with the control group, the inhibition rate of SiHa cells treated by 5.0 μg/mL dioscin for 24 h was 53.62% (Figure 2B). Thus, the SiHa cells treated by dioscin at the concentrations of 1.25, 2.5 and 5.0 µg/mL for 24 h were selected for subsequent experiments.

Under these conditions, the action of dioscin on normal cervical epithelial H8 cells was examined too (Figure S1). The inhibition rates of H8 cells treated by dioscin (5.0 µg/mL) for 12 h and 24 h were 19.96% and 25.55%, suggesting that dioscin caused slight cytotoxic effects in normal cells.

### 2.2. Dioscin-Induced Cells Ultrastructure Changes

As shown in Figure 2C,D, the HeLa and SiHa cells in the control groups exhibited typical normal ultra structures, including round nuclei, sharp edges and complete nuclear membranes, while dioscin-treated HeLa cells displayed the typical features of apoptosis, including nuclear chromatin condensation and marginalization (red arrow, 8000× in the left). Meanwhile, compared with control groups, dioscin caused mitochondrial double membrane decomposition, along with mitochondrial swelling and cristae fracture.

### 2.3. Dioscin-Induced Morphological Changes

**Figure 3:**
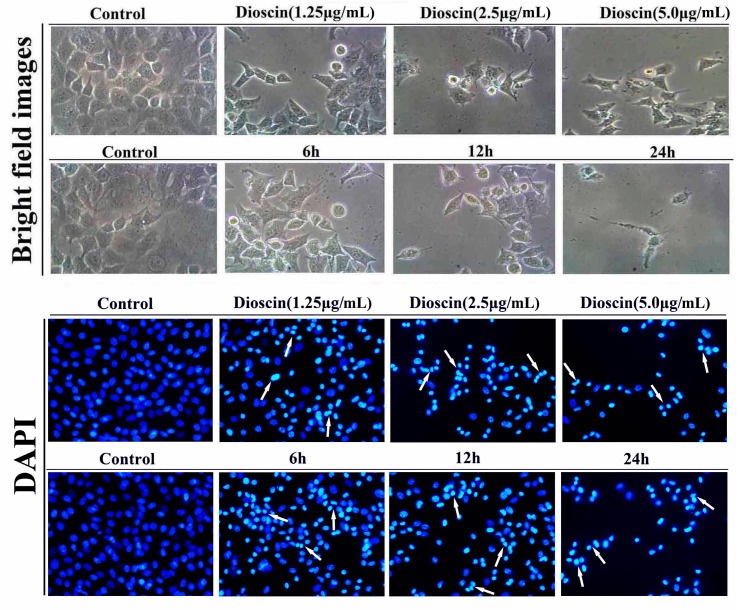
Morphological changes of HeLa cells treated by dioscin.

**Figure 4:**
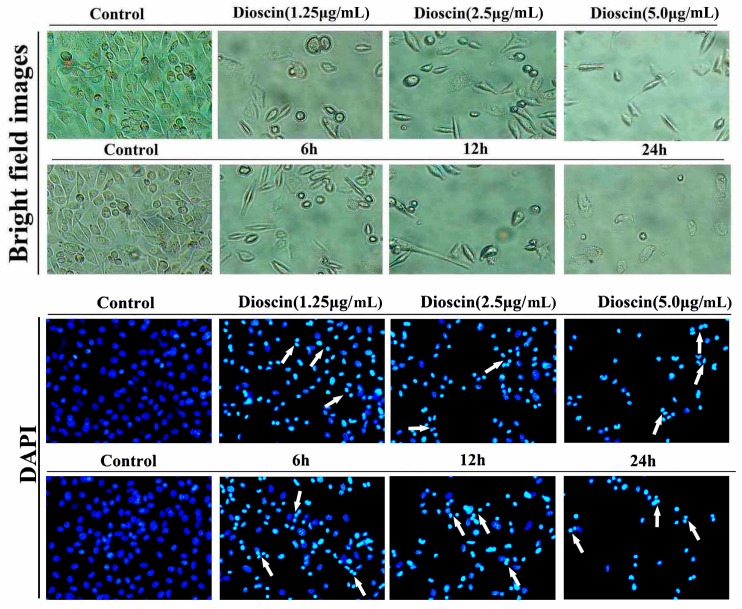
Morphological changes of SiHa cells treated by dioscin.

The morphological changes of HeLa and SiHa cellstreated by dioscin were investigated based on DAPI staining (200×, final magnification). As shown in Figure 3 and Figure 4, the nucleus of the cancer cells was condensed, and the nuclear apoptotic bodies were formed and brighter white arrow). The results indicated that dioscin strongly inhibited the proliferation of HeLa and SiHa cells.

### 2.4. Effects of Dioscin on ROS Levels

**Figure 5:**
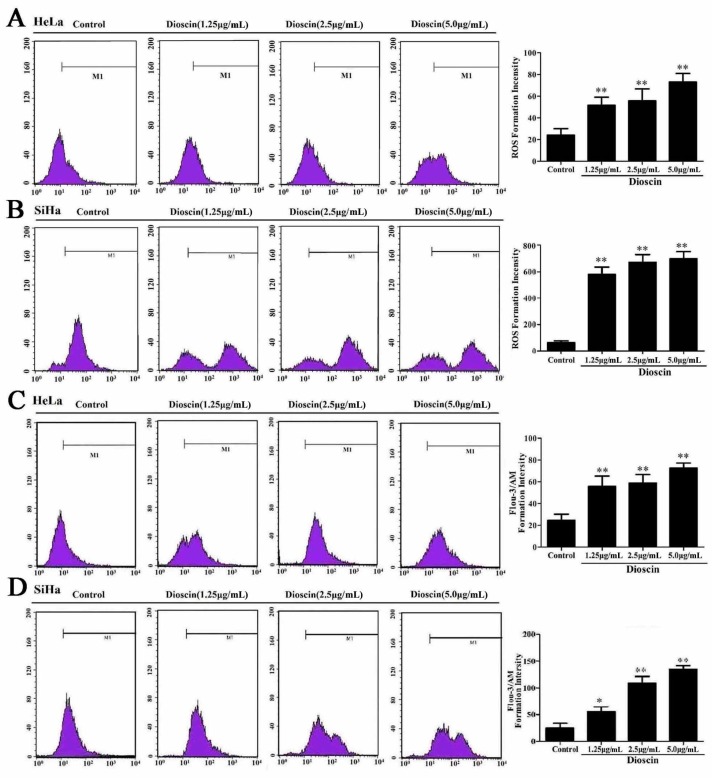
Effects of dioscin on ROS levels and Ca^2+^ release. (**A**) ROS generation in HeLa cell treated by dioscin; (**B**) ROS generation in SiHa cell treated by dioscin; (**C**) Effects of dioscin on Ca^2+^ level in HeLa cells by flow cytometry; (**D**) Effects of dioscin on Ca^2+^ level in SiHa cells by flow cytometry. Data are presented as mean ± SD (*n* = 5). * *p* < 0.05 and ** *p* < 0.01 compared with control group.

Dioscin significantly affected ROS generation in HeLa and SiHa cells. As shown in Figure 5A,B, after being treated with dioscin (1.25, 2.5 and 5.0 μg/mL), the ROS levels of the cancer cells were notably increased.

### 2.5. Dioscin Caused Ca^2+^ Release in HeLa and SiHa Cells

As shown in Figure 5C, in HeLa cells, after being treated with different concentrations of dioscin for 12 h, the levels of Ca^2+^ were obviously increased. As shown in Figure 5D, in SiHa cells, after being treated with dioscin (1.25, 2.5 and 5.0 μg/mL) for 24 h, the levels of Ca^2+^ were also notably increased.

### 2.6. Dioscin Induced DNA Damage of the HeLa and SaHa Cells

**Figure 6:**
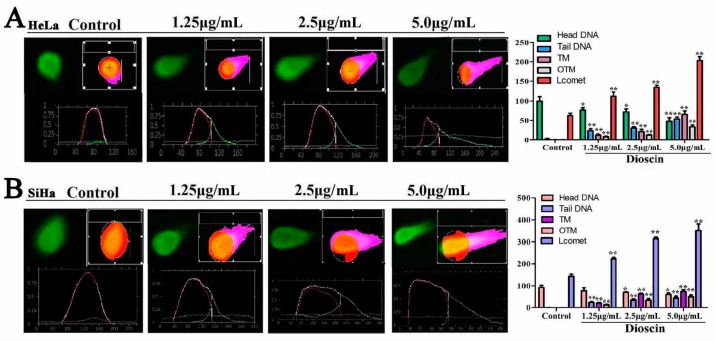
Dioscin induced DNA damage and cell cycle arrest of the HeLa and SaHa cells. (**A**) Dioscin induced DNA damage in HeLa cells by SCGE assay (200×, final magnification); (**B**) Dioscin induced DNA damage in SiHa cells by SCGE assay (200×, final magnification). Data are presented as mean ± SD (*n* = 3). * *p* < 0.05 and ** *p* < 0.01 compared with control group.

When the HeLa cells were treated by dioscin, the DNA fragment migration formed smears with a small head and big tail, and the analyzed curves were inserted inside each picture (Figure 6A). The non-apoptotic cells showed single peaks (head DNA), but the apoptotic cells were double peaks (head DNA and tail DNA).The length of the DNA migration smears (comet tails) was markedly increased, and the head DNA was significantly decreased. When the SaHa cells were treated by dioscin, the results were the same as for HeLa cells (Figure 6B).

### 2.7. Dioscin Affects theMitochondrialSignaling Pathway

**Figure 7:**
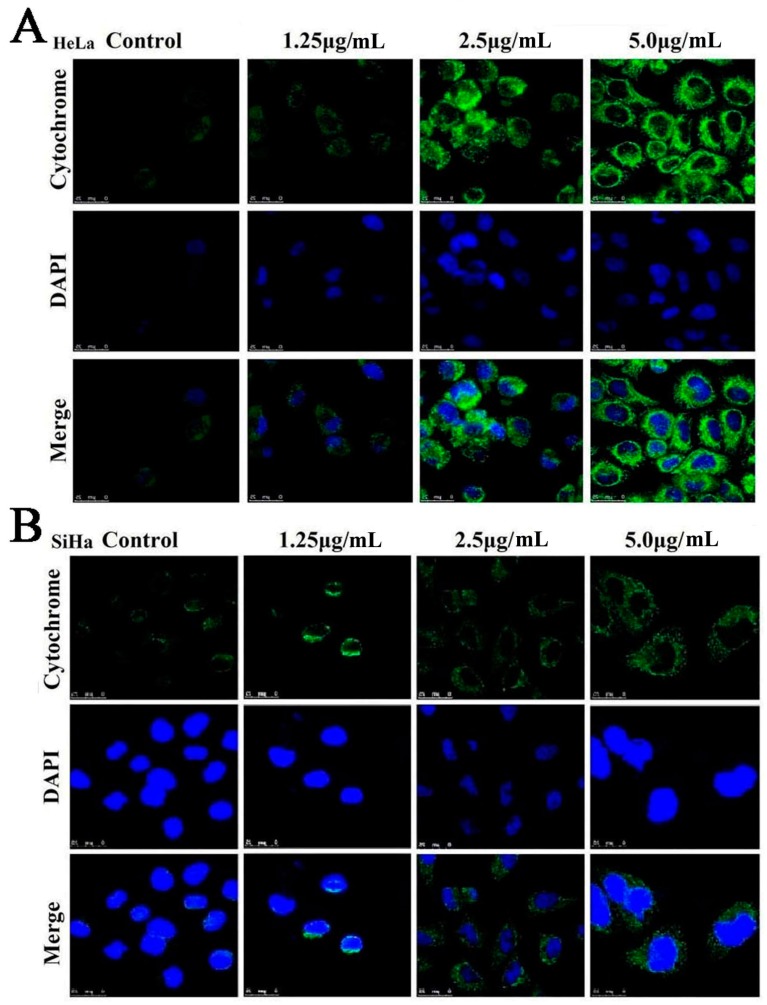
(**A**) Effects of dioscin on cytochrome C release in Hela cells (800×, final magnification); (**B**) Effects of dioscin on cytochrome C release in SiHa cells (800×, final magnification).

As shown in Figure 7A,B, the HeLa and SiHa cells in control groups showed a point or massive staining pattern, while the staining of the cells treated by dioscin (5.0 μg/mL) was diffuse and had a bright green light glow, which indicated that dioscin caused the release of cytochrome C from the mitochondria into the cytosol.

### 2.8. Dioscin Induced HeLa and SiHa Cell Apoptosis

**Figure 8:**
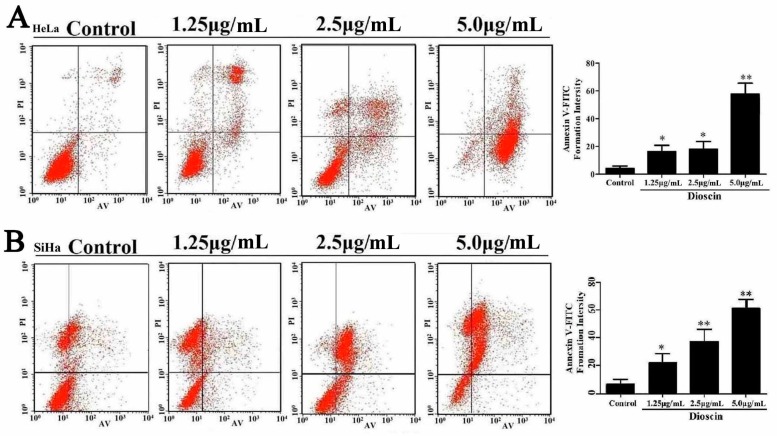
Dioscin induced HeLa and SiHa cell apoptosis. (**A**) Dioscin caused apoptosis in HeLa cells by flow cytometric analysis with Annexin V-FITC and PI-staining; (**B**) Dioscin caused apoptosis in SiHa cells by flow cytometric analysis with Annexin V-FITC and PI-staining. Data are presented as mean ± SD (*n* = 3). * *p*< 0.05 and ** *p*< 0.01 compared with control group.

**Figure 9:**
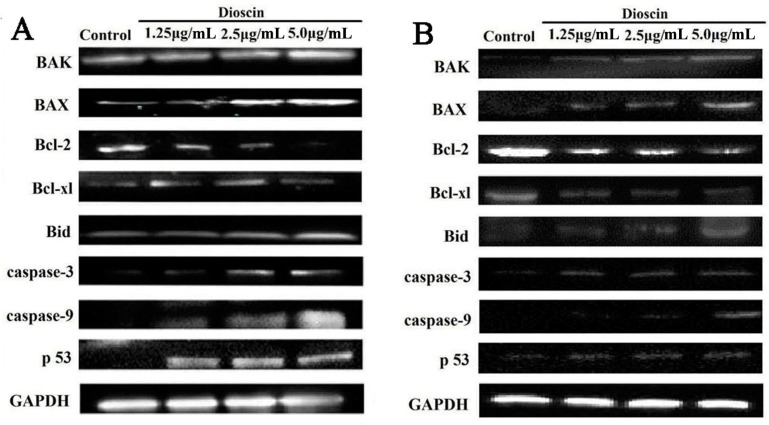
Dioscin adjusted cell apoptosis signaling pathway. (**A**) Effects of dioscin on the protein levels of Bcl-2, Bcl-xl, Bax, Bak, Bid, p53, caspase-3 and caspase-9 in HeLa cell; (**B**) Effects of dioscin on the protein levels of Bcl-2, Bcl-xl, Bax, Bak, Bid, p53, caspase-3 and caspase-9 in SiHa cell.

In this work, Annexin V/PI binding was used to evaluate the apoptosis of the cancer cells induced by dioscin. As shown in Figure 8A, the percentages of apoptotic cells were significantly increased from 3.92% ± 2.09% to 22.86% ± 3.01%, 23.68% ± 4.5% and 66.03% ± 4.51% after the HeLa cells were treated with dioscin (1.25, 2.5 and 5.0 μg/mL) for 12 h. In addition, the percentages of apoptotic cells were significantly increased from 10.33% ± 6.39% to 38.92% ± 12.39%, 47.13% ± 13.61% and 56.99% ± 14.24% after the SiHa cells treated with dioscin (1.25, 2.5 and 5.0 μg/mL) for 24 h (Figure 7B). As shown in Figure 9A,B, dioscin significantly down-regulated the expression levels of the anti-apoptotic proteins including Bcl-2 and Bcl-xl, compared with control groups. Meanwhile, the expression levels of Bax, Bak, Bid, p53 caspase-3 and -9 were significantly up-regulated by dioscin.

## 3. Discussion

Cervical carcinoma, one of the most common malignancies, represents a significant threat to women’s health and there is an urgent need to develop new anti-cancer drugs for its treatment. HeLa cells are aHPV18 DNA-positive cervical cancer cell line. It can express HPV18 E6 and E7 proteins, which play key roles in maintaining the malignant cervical cancer phenotype. In addition, the SiHa cell line is a kind of malignant tumor that integrates with the HPV16 gene. The early cancer gene E6 plays an important promoting effect in the malignant transformation of cervical epithelial cells [16,17,18]. Thus, the biologies of HeLa and SiHa cells may be relevant to the expression of HPV-induced tumor proteins.

Dioscin is a steroidal saponin. Previous studies have shown that dioscin processes potent activities against breast cancer and prostate carcinoma [19,20]. However, these studies always focused on the effects of dioscin-induced cell apoptosis. As far as we know, ROS, Ca^2+^, DNA damage and cytochrome C also play critical roles in cancer progression. Our previous work also found that dioscin had anti-gastric cancer action via induction of cell apoptosis, DNA damage, mitochondrial structure changes, ROS and Ca^2+^ generation, cell cycle arrest and cell migration [21]. Up to now, we already know that dioscin can induce the apoptosis of HeLa cells. However, to our best knowledge, there have no published papers to report the anti-cancer effects of dioscin against SiHa cells, and the mechanisms of the action on cervical cancer are still unknown. Therefore, the aim of the present study was further to confirm the effects of dioscin against HeLa and SiHa cells *in vitro*, and then to investigate the possible mechanism(s) of action.

The experiments indicated the dioscin inhibited the viability of HeLa and SaHa cells and induced morphological changes. The MTT results showed that the proliferation of HeLa and SiHa cells was significantly inhibited by dioscin in atime- and dose-dependent manner. In order to determine the toxic effect of this compound on normal cells in carcinoma-adjacent tissue under the same conditions, we examined the action of dioscin on the HPV16 immortalized human cervical mucosa epithelial H8 cell line. The MTT results showed that the rate of inhibition ofH8 cells treated with dioscin (5.0 µg/mL) for 24 h was 25.55%, which was significantly less than the inhibition rates of HeLa (80%) and SiHa (53.62%) cells. Dioscin at the dose of 5.0 µg/mL caused slight cytotoxic effects on normal cells. Therefore, these results indicate that doscin maybe a highly effective and low toxicity drug to treat cervical carcinoma.

Recently, the anti-cancer effects of ROS, which can affect mitochondrial membrane potential and membrane permeability, have been recognized, [22,23]. Massive accumulation of intracellular ROS is regarded as a signal to initiate apoptosis, which can trigger a series of mitochondria- associated events [4]. Simultaneously, accumulation of excessive ROS can lead to lipid peroxidation, protein oxidation, enzyme inactivation and oxidative DNA damage [24,25,26]. Thus, ROS is considered as an important target for the treatment of cervical carcinoma [27]. Generally, the three kinds of proteins associated with apoptosis can be divided into anti-apoptotic, pro-apoptotic and BH3-only proteins [28]. Bcl-2 family is a group of important regulators of apoptosis in the mitochondrial apoptosis pathway. The anti-apoptotic proteins Bcl-2 and Bcl-xl can suppress apoptosis through preventing the formation of mitochondrial pores, protecting membrane integrity, and inhibiting release of cytochrome C [29]. Bax, a pro-apoptotic protein, can enter the mitochondria and trigger apoptosis, and cause the release of cytochrome C [30]. In addition, the ratio of Bcl-2/Bax is an important indicator that can induce the apoptosis progress of cancer cells. The BH3-only subseries include Bid, Bik, Bim and Bad. The crosstalk between cell surface receptor-mediated apoptotic signals and mitochondria can be mediated by Bid [31]. Cytochrome C can activate caspase protein and stimulate apoptosis [32]. In the present work, the results showed that dioscin significantly down-regulated the protein levels of Bcl-2 and Bcl-xl, and up-regulated the protein levels of Bax, Bak, Bid, p53, caspase-3 and -9, suggesting that regulating mitochondria signaling pathway may be one potential mechanism of action of dioscin against HeLa and SiHa cells.

## 4. Materials and Methods

### 4.1. Materials, Cell Culture and Viability Assay

Dioscin with a purity of >98% was obtained from the National Institute for the Control of Pharmaceutical and Biological Products (Beijing, China). The human cervical carcinoma HeLa and SiHa cells were provided by Shanghai Cell Biology Institute of Chinese Academy of Sciences (Shanghai, China). The normal cervical epithelial H8 cells were obtained from Berthold (Shanghai, China). The cells were cultured in RPMI-1640 medium with low carbohydrates containing 10% fetal bovine serum (FBS) at 37 °C in 5% CO_2_.The HeLa, SiHa and H8 cells were plated in 96-well plates at the density of 1 × 10^5^ cells/well and cultured for 24 h, and then treated with different concentrations of dioscin (1.25, 2.5 and 5.0 μg/mL) under different times (6, 12 and 24 h) for MTT assay. The absorbance was measured at a wavelength of 570 nm using a microplate reader (Thermo, Waltham, MA, USA).

### 4.2. Transmission Electron Microscope (TEM) Assay

The HeLa and SiHa cells (2 × 10^5^ cells/mL) were plated in 6-well plates, treated with dioscin, harvested, and fixed overnight at 4 °C in 2% glutaraldehyde. The samples were implemented as previously described [27]. The obtained sections were then stained and observed using a transmission electron microscope (JEM-2000EX, JEOL, Tokyo, Japan).

### 4.3. DAPI Staining

HeLa and SiHa cells were seeded in six-well plates and cultured overnight, then treated separately with dioscin (1.25, 2.5 and 5.0 μg/mL) for 12 h and 24 h. For DAPI staining, the cells were treated as mentioned above, and then stained with DAPI (1.0 μg/mL) solution. Finally, the images were photographed with a fluorescence microscope (Olympus, Tokyo, Japan).

### 4.4. Detection of Intracellular ROS Accumulation

The HeLa and SiHa cells were plated in 6-well plates at the density of 1 × 10^5^ cells/well and treated by dioscin(1.25, 2.5 and 5.0 μg/mL). The cells were collected and re-suspended in 500 μL DCFH-DA (10.0 μM), which were all analyzed by flow cytometry (AccuriC6, Becton-Dickinson, Lake Franklin, NJ, USA).

### 4.5. Detection of Cell Apoptosis and Intracellular Ca^2+^Release

HeLa and SiHa cells were plated in 6-well plates at the density of 1 × 10^5^ cells/well and treated with dioscin (1.25, 2.5 and 5.0 μg/mL), then collected and resuspended in 500 μL of Fluo-3/AM (2.5 μM), which were all analyzed by flow cytometry (Becton-Dickinson).

### 4.6. Single Cell Gel Electrophoresis Assay

After the cancer cells being treated with dioscin (1.25, 2.5 and 5.0 μg/mL), single cell gel electrophoresis (SCGE) method was used to detect dioscin-induced DNA damage. The images of the cells were obtained by a fluorescence microscope (Olympus) according to the manufacturer’s instructions (Cell Biolabs, Inc., San Diego, CA, USA). Eventually, over 50 cells were randomly selected from each of the three repeated wells and analyzed by the Comet Assay Software Project (CASP) 1.2.2.

### 4.7. Detection of Cytochrome C Release

HeLa and SiHa cells were seeded in six-well plates, treated with dioscin (1.25, 2.5 and 5.0 μg/mL), and then incubated with the primary antibody overnight. After that, the plates were incubated with the secondary antibody for 1 h at 37 °C, and dyed with DAPI (5.0 μg/mL) for 5 min. The images of the cells were obtained by a laser scanning confocal microscope (TCS SP5, Leica, Germany).

### 4.8. Detection of Cell Apoptosis

Cell apoptosis was analyzed by flow cytometry based on the Annexin V-FITC/propidium iodide apoptosis kit (Beyotime, Shanghai, China). Briefly, the HeLa cells and SiHa cells c were treated by dioscin (1.25, 2.5 and 5.0 μg/mL), then collected and washed twice by ice-cold PBS. After that, the cells were stained with Annexin V-FITC/propidium iodide. Apoptosis was evaluated by a flow cytometry assay (Becton-Dickinson).

### 4.9. Western Blotting Assay

HeLa and SiHa cells were treated with dioscin (1.25, 2.5 and 5.0 μg/mL), and protein samples were extracted using cell lysis buffer (Beyotime, Shanghai, China). An aliquot (50 μg protein) was loaded onto a 8%–12% SDS-PAGE gel and separated electrophoretically. Then the target proteins were transferred to a PVDF membrane (Millipore, MA, USA). After blocking in 5% dried skim milk (Boster Biological Technology, Wuhan, China), the PVDF membrane was incubated with primary antibodies (listed in Table S1), respectively. Then the membrane was incubated with horseradish peroxidase-conjugated antibodies at a 1:5000 dilution. Protein detection was performed based on an enhanced chemiluminescence (ECL) method and photographed by using a BioSpectrum Gel Imaging System (HR410, UVP, Upland, CA, USA). In order to eliminate the variations, data were adjusted to GAPDH expression: IOD of objective protein versus IOD of GAPDH expression.

### 4.10. Statistical Analysis

Unless otherwise stated, all the data were analyzed by using the statistical software SPSS 11.5, expressed as means ± SD and analyzed statistically by one-way analysis of variance (ANOVA). A value of *p* < 0.05 was considered statistically significant.

## 5. Conclusions

Dioscin induced apoptosis of human cervical cancer HeLa and SiHa cells through inducing ROS-mediated DNA damage and activating the mitochondrial signaling pathway. However, based on the *in vitro* results, the molecular mechanism of action and the drug targets of dioscin need further investigation.

## Supplementary Materials

Supplementary materials can be accessed at: http://www.mdpi.com/1420-3049/21/6/730/s1.

## References

[1] Jemal A., Bray F., Center M.M., Ferlay J., Ward E., Forman D. (2011). Global cancer statistics. CA Cancer J. Clin..

[2] Hu M., Xu L., Yin L., Qi Y., Li H., Xu Y., Han X., Peng J., Wan X. (2013). Cytotoxicity of dioscin in human gastric carcinoma cells through death receptor and mitochondrial pathways. J. Appl. Toxicol..

[3] Liu S., Yuan Y., Okumura Y., Shinkai N., Yamauchi H. (2010). Camptothecin disrupts androgen receptor signaling and suppresses prostate cancer cell growth. Biochem. Biophys. Res. Commun..

[4] Pathak N., Khandelwal S. (2007). Role of oxidative stress and apoptosis in cadmium induced thymic atrophy and splenomegaly in mice. Toxicol. Lett..

[5] Parent N., Winstall E., Beauchemin M., Paquet C., Poirier G.G., Bertrand R. (2009). Proteomic analysis of enriched lysosomes at early phase of camptothecin-induced apoptosis in human U-937 cells. J. Proteom..

[6] Jiang S., Fan J., Wang Q., Ju D., Feng M., Li J., Guan Z.B., An D., Wang X., Ye L. (2016). Diosgenin induces ROS-dependent autophagy and cytotoxicity via mTOR signaling pathway in chronic myeloid leukemia cells. Phytomedicine.

[7] Liang Y., Li X., He X., Qiu X., Jin X., Zhao X.Y., Xu R.Z. (2016). Polyphyllin I induces cell cycle arrest and apoptosis in human myeloma cells via modulating β-catenin signalling pathway. Eur. J. Haematol..

[8] Zhang C., Jia X., Bao J., Chen S., Wang K., Zhang Y., Li P., Wan J.B., Su H., Wang Y. (2016). Polyphyllin VII induces apoptosis in HepG2 cells through ROS-mediated mitochondrial dysfunction and MAPK pathways. BMC Complement. Altern. Med..

[9] Jin J., Jin X., Qian C., Ruan Y., Jiang H. (2013). Signaling network of OSW-1-induced apoptosis and necroptosis in hepatocellular carcinoma. Mol. Med. Rep..

[10] Li M., Han X., Yu B. (2003). Synthesis of monomethylated dioscin derivatives and their antitumor activities. Carbohydr. Res..

[11] Ikeda T., Ando J., Miyazono A., Zhu X.H., Tsumagari H., Nohara T., Yokomizo K., Uyeda M. (2000). Anti-herpes virus activity of Solanum steroidal glycosides. Biol. Pharm. Bull..

[12] Lu B., Yin L., Xu L., Peng J. (2011). Application of proteomic and bioinformatic techniques for studying the hepatoprotective effect of dioscin against CCl(4)-induced liver damage in mice. Planta Med..

[13] Tao X., Wan X., Xu Y., Xu L., Qi Y., Yin L., Han X., Lin Y., Peng J. (2014). Dioscin attenuates hepatic ischemia-reperfusion injury in rats through inhibition of oxidative-nitrative stress, inflammation and apoptosis. Transplantation.

[14] Wei Y., Xu Y., Han X., Qi Y., Xu L., Xu Y., Yin L., Sun H., Liu K., Peng J. (2013). Anti-cancer effects of dioscin on three kinds of human lung cancer cell lines through inducing DNA damage and activating mitochondrial signal pathway. Food Chem. Toxicol. Int. J. Publ. Br. Ind. Biol. Res. Assoc..

[15] Cai J., Liu M., Wang Z., Ju Y. (2002). Apoptosis induced by dioscin in Hela cells. Biol. Pharm. Bull..

[16] Franco E.L. (1996). Epidemiology of anogenital warts and cancer. Obstet. Gynecol. Clin. N. Am..

[17] Walboomers J.M., Jacobs M.V., Manos M.M., Bosch F.X., Kummer J.A., Shah K.V. (1999). Human papillomavirus is a necessary cause of invasive cervical cancer worldwide. J. Pathol..

[18] Wright J.D., Herzog T.J. (2002). Human papillomavirus: emerging trends in detection and management. Curr. Womens Health Rep..

[19] Kim E.A., Jang J.H., Lee Y.H., Sung E.G., Song I.H., Kim J.Y., Kim S., Sohn H.Y., Lee T.J. (2014). Dioscin induces caspase-independent apoptosis through activation of apoptosis-inducing factor in breast cancer cells. Apoptosis.

[20] Chen J., Li H.M., Zhang X.N., Xiong C.M., Ruan J.L. (2014). Dioscin-induced apoptosis of human LNCaP prostate carcinoma cells through activation of caspase-3 and modulation of Bcl-2 protein family. J. Huazhong Univ. Sci. Technol. Med. Sci..

[21] Zhao X., Xu L., Zheng L., Yin L., Qi Y., Han X., Xu Y., Peng J. (2016). Potent effects of dioscin against gastric cancer *in vitro* and *in vivo*. Phytomedicine.

[22] Chatterjee S., Kundu S., Bhattacharyya A. (2008). Mechanism of cadmium induced apoptosis in the immunocyte. Toxicol. Lett..

[23] Martin K.R., Barrett J.C. (2002). Reactive oxygen species as double-edged swords in cellular processes: Low-dose cell signaling versus high-dose toxicity. Hum. Exp. Toxicol..

[24] Green D.R., Reed J.C. (1998). Mitochondria and apoptosis. Science (New York, N. Y.).

[25] Mallis R.J., Buss J.E., Thomas J.A. (2001). Oxidative modification of H-ras: S-thiolation and S-nitrosylation of reactive cysteines. Biochem. J..

[26] Simon H.U., Haj-Yehia A., Levi-Schaffer F. (2000). Role of reactive oxygen species (ROS) in apoptosis induction. Apoptosis Int. J. Programm. Cell Death.

[27] Schumacker P.T. (2006). Reactive oxygen species in cancer cells: live by the sword, die by the sword. Cancer Cell.

[28] Borner C. (2003). The Bcl-2 protein family: Sensors and checkpoints for life-or-death decisions. Mol. Immunol..

[29] Brenner C., Cadiou H., Vieira H.L., Zamzami N., Marzo I., Xie Z., Leber B., Andrews D., Duclohier H., Reed J.C. (2000). Bcl-2 and Bax regulate the channel activity of the mitochondrial adenine nucleotide translocator. Oncogene.

[30] Vyssokikh M.Y., Zorova L., Zorov D., HeimLich G., Jurgensmeier J.J., Brdiczka D. (2002). Bax releases cytochrome c preferentially from a complex between porin and adenine nucleotide translocator. Hexokinase activity suppresses this effect. Mol. Biol. Rep..

[31] Luo X., Budihardjo I., Zou H., Slaughter C., Wang X. (1998). Bid, a Bcl2 interacting protein, mediates cytochrome c release from mitochondria in response to activation of cell surface death receptors. Cell.

[32] Huttemann M., Pecina P., Rainbolt M., Sanderson T.H., Kagan V.E., Samavati L., Doan J.W., Lee I. (2011). The multiple functions of cytochrome c and their regulation in life and death decisions of the mammalian cell: From respiration to apoptosis. Mitochondrion.

