# Correction: Zhao, X.W., et al. Dioscin Induces the Apoptosis of Human Cervical Carcinoma HeLa and SiHa Cells Through ROS-mediated DNA Damage, Cell Cycle Arrest and Mitochondrial Signaling Pathways. *Molecules* 2016, *21*, 730

**DOI:** 10.3390/molecules25010232

**Published:** 2020-01-06

**Authors:** Xinwei Zhao, Xufeng Tao, Lina Xu, Lianhong Yin, Yan Qi, Youwei Xu, Xu Han, Jinyong Peng

**Affiliations:** College of Pharmacy, Dalian Medical University, Western 9 Lvshunnan Road, Dalian 116044, China; zhaoxinwei2015dy@163.com (X.Z.); taoxufeng@dmu.edu.cn (X.T.); Linaxu_632@126.com (L.X.); Lianhongyin_1980@163.com (L.Y.); Yanqi_1976@163.com (Y.Q.); Youweixu_1964@163.com (Y.X.); Xuhan2002zs@163.com (X.H.)

During the course of a review of our publications, an error in the title paper [1] has come to our attention. This error affects the flow cytometry data presented in Figure 8. We provide below, the correct figure. The data have been reanalyzed and determined to have no influence on the reported results. 

All co-authors agree with the content of this correction and wish to apologize for any inconvenience to the readers resulting from this error.

## Figures and Tables

**Figure 8:**
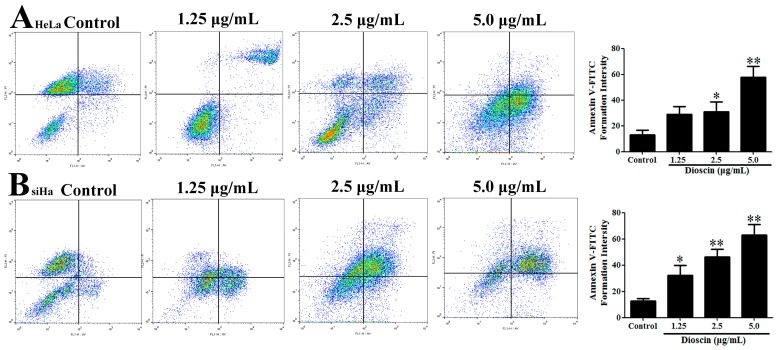
(**A**) Dioscin-caused apoptosis in HeLa cells by flow cytometric analysis with Annexin V-FITC and PI-staining; (**B**) Dioscin-caused apoptosis in SiHa cells by flow cytometric analysis with Annexin V-FITC and PI-staining. Data are presented as mean ± SD (*n* = 3). * *p* < 0.05 and ** *p* < 0.01 compared with control group.
