# Supplementary material for: Dioscin Induces Apoptosis in Human Cervical Carcinoma HeLa and SiHa Cells through ROS-Mediated DNA Damage and the Mitochondrial Signaling Pathway
Source: Molecules. 2016 Jun 4;21(6):730. doi: 10.3390/molecules21060730 (PMC6273920; doi:10.3390/molecules21060730)
Supplement: Supplementary file 1 [file molecules-21-00730-s001.pdf]

Supplementary Materials: Dioscin Induces Apoptosis in Human Cervical Carcinoma HeLa and SiHa Cells through ROS-Mediated DNA Damage and the Mitochondrial Signaling Pathway

Xinwei Zhao, Xufeng Tao, Lina Xu, Lianhong Yin, Yan Qi, Youwei Xu, Xu Han and Jinyong Peng

Table S1. The information of the antibodies used in the present work.

Antibody *	Dilution ratio
Bcl-2	(1: 1000 dilution)
Bcl-xl	(1: 1000 dilution)
Bax	(1: 1000 dilution)
Bak	(1: 1000 dilution)
Bid	(1: 500 dilution)
p53	(1: 1000 dilution)
Caspase-3	(1: 1000 dilution)
Caspase-9	(1: 1000 dilution)
GAPDH	(1: 2000 dilution)

* Manufacturer: Proteintech Group, Inc. Chicago, IL, USA.

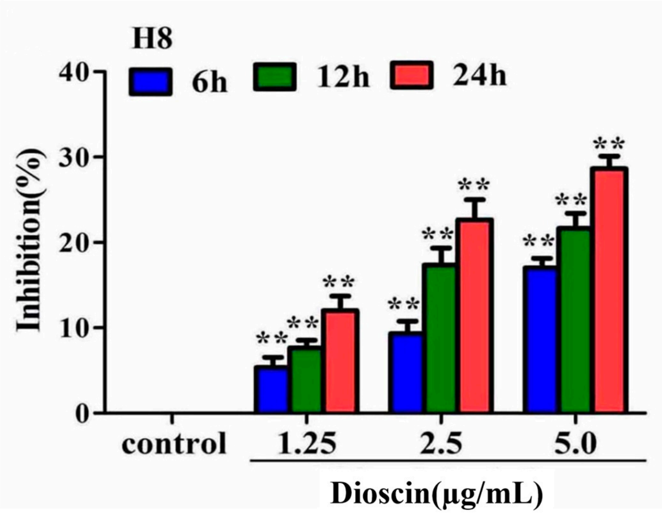

Figure S1. Inhibition effects of dioscin on H8 cells. Data are presented as mean ± SD ($n = 5$). * $p < 0.05$ and ** $p < 0.01$ compared with control group.
